# Malaria community health workers in Myanmar: a cost analysis

**DOI:** 10.1186/s12936-016-1102-3

**Published:** 2016-01-25

**Authors:** Shwe Sin Kyaw, Tom Drake, Aung Thi, Myat Phone Kyaw, Thaung Hlaing, Frank M. Smithuis, Lisa J. White, Yoel Lubell

**Affiliations:** Mathematical and Economic Modelling, Mahidol-Oxford Tropical Medicine Research Unit, 420/6 Rajvithi Rd, Bangkok, 10400 Thailand; Centre for Tropical Medicine, Nuffield Department of Medicine, University of Oxford, Oxford, UK; Department of Public Health, Ministry of Health, Nay Pyi Taw, Myanmar; Department of Medical Research, Ministry of Health, Yangon, Myanmar; Medical Action Myanmar, Yangon, Myanmar; Myanmar Oxford Clinical Research Unit, Yangon, Myanmar

**Keywords:** Community health worker, Malaria, Cost, Cost analysis, Economic evaluation

## Abstract

**Background:**

Myanmar has the highest malaria incidence and attributed mortality in South East Asia with limited healthcare infrastructure to manage this burden. Establishing malaria Community Health Worker (CHW) programmes is one possible strategy to improve access to malaria diagnosis and treatment, particularly in remote areas. Despite considerable donor support for implementing CHW programmes in Myanmar, the cost implications are not well understood.

**Methods:**

An ingredients based micro-costing approach was used to develop a model of the annual implementation cost of malaria CHWs in Myanmar. A cost model was constructed based on activity centres comprising of training, patient malaria services, monitoring and supervision, programme management, overheads and incentives. The model takes a provider perspective. Financial data on CHWs programmes were obtained from the 2013 financial reports of the Three Millennium Development Goal fund implementing partners that have been working on malaria control and elimination in Myanmar. Sensitivity and scenario analyses were undertaken to outline parameter uncertainty and explore changes to programme cost for key assumptions.

**Results:**

The range of total annual costs for the support of one CHW was US$ 966–2486. The largest driver of CHW cost was monitoring and supervision (31–60 % of annual CHW cost). Other important determinants of cost included programme management (15–28 % of annual CHW cost) and patient services (6–12 % of annual CHW cost). Within patient services, malaria rapid diagnostic tests are the major contributor to cost (64 % of patient service costs).

**Conclusion:**

The annual cost of a malaria CHW in Myanmar varies considerably depending on the context and the design of the programme, in particular remoteness and the approach to monitoring and evaluation. The estimates provide information to policy makers and CHW programme planners in Myanmar as well as supporting economic evaluations of their cost-effectiveness.

## Background

Myanmar has the highest malaria incidence and attributed mortality in southeast Asia [1]. Historically, southeast Asia has been the epicentre of malaria drug resistance and resistance to artemisinin, the foundation of antimalarial treatment globally, has spread in East and much of upper Myanmar [2–4]. Malaria services are delivered by a wide range of governmental and non-governmental organisations as well as the private sector. Approximately 70 % of the population in Myanmar reside in rural areas [5]. A key challenge to effective malaria control in these settings is the limited healthcare infrastructure. Where health facilities have been upgraded staffing them with well-trained healthcare providers remains a challenge. Government health services reach larger towns and villages but many of the most remote smaller villages have no coverage of health services.

In low income countries training and support of Community Health Workers (CHWs) has been identified as one possible strategy to address the shortage of basic health services in areas where accessibility to health centres is limited. Malaria CHWs are trained to diagnose febrile patients with malaria rapid diagnostic test (RDT) and provide them with anti-malarial medication after parasitological confirmation. This aims to treat patients within 24 h of onset of fever while reducing unnecessary use of anti-malarial medication.

CHW programmes were found to be effective [6, 7] and cost-effective [8] in different settings and different health areas [9, 10]. A cluster randomized trial conducted in 2012 demonstrated modest improvements to malaria healthcare in Myanmar following the introduction of malaria CHWs [6] and a study related to this one has examined cost effectiveness and resource allocation scenarios of insecticide treated bed nets and malaria CHWs in Myanmar [11]. With the support of international donors, the NMCP and implementing partners are expanding the CHWs programme. The cost of implementing CHW programmes in Myanmar, however, is not well established. Estimating the full cost of a CHWs programme is an important component of policy planning. This study estimates annual cost of CHWs given different programmatic options and identifies key cost-drivers, with the aim of helping policy makers plan malaria CHWs programmes in Myanmar.

## Methods

### Community health workers for malaria in Myanmar

CHWs are supposed to be nominated by village health committee. As village health committees are often not established or functioning, candidates for CHWs are often suggested by the village leader and the organization that supports the CHW or an NGO makes the final selection. The selection is usually based on criteria including education level and position in the community. Candidates are often individuals that already provide informal health care in their community. CHWs can be of any gender and aged between 18 and 50 years. They should have sufficient education to read and write Myanmar (Burmese) language and preferably speak local dialects.

Malaria CHWs are trained to obtain simple history of malaria symptoms and use an RDT to screen for parasitaemia. Patients with a positive RDT are treated with the appropriate anti-malarial. Severe and complicated malaria patients are referred to the nearest hospital, and CHWs are instructed to report immediately to the health centre when there are unusual high occurrences of malaria or febrile patients in the community. In addition to malaria diagnosis and treatment they typically also provide health education and assist health staff during impregnation sessions of bed nets or the distribution of long-lasting insecticidal nets (LLINs) in the village. Some organizations have extended the role of their CHWs beyond malaria control to other diseases and interventions, implying additional costs and potentially higher health benefits. While such diversification can improve the efficiency of CHW programmes [11], in this analysis the focus is restricted to their role in improving malaria diagnosis and treatment, and their cost is attributed fully to this aim.

### Costing

An ingredients based micro-costing approach was used to develop a model of the annual implementation cost of a malaria CHW in Myanmar whereby all ‘ingredients’ necessary to implement a CHW programme are (1) identified, (2) measured and (3) valued. The cost model was constructed based on the activity cost centres for CHWs comprising training, patient services, monitoring and supervision, programme management, overheads and incentives. The model takes a provider perspective to help inform funders and programme managers on resource allocation and project planning. Costs to patients are excluded but considered to be very low since the CHWs are based in their community and services are usually free of charge.

A key aspect of this analysis was to consider how the cost of CHW programmes differs in more or less remote contexts. To this end, four remoteness strata were defined; easy, medium, difficult and very difficult to access. These strata are defined by incremental increases in travel costs and a change in the mode of monitoring and supervision; details below. The model is based on a set of key parameters, detailed in Table 1. Model parameters can be adjusted to observe the effect of programme variation or parameter uncertainty on programme costs.

**Table 1:** Community health worker costing model parameters

Parameters	Model input	Lower value	Upper value
Economic			
Exchange rate	810	699	1000
Discount rate	3 %	0 %	8 %
Training			
Number of trainers	2	1	3
Number of facilitators	1	1	3
Number of CHWs in initial training	25	10	40
Number of days for initial training	5	3	7
Number of CHWs active year	3	1	5
Number of CHWs in refresher training	55	30	90
Frequency of refresher training per year	1	0.5	2
Patient services			
Number of tests performed by CHW per year	135	20	250
Percentage of test positivity	14 %	1 %	50 %
Test price	0.69	0.48	0.90
Village size	500	100	671
Monitoring and supervision			
Number of field supervision per CHW*	8	4	12
Number of meetings in health centre per year**	12	8	12
Percentage of attendant in monthly meetings**	80 %	50 %	100 %
CHWs incentive			
Monthly incentive	20	5	50
Incentive for negative test	0.2	0.2	0.5
Incentive for positive test	0.3	0.3	1
Overhead	10 %		

* Field supervision (remote settings)

** Health centre supervision (accessible settings)

Financial data on CHWs programmes were obtained from the 2013 financial reports of the Three Millennium Development Goal fund (3MDG) implementing partners that have been working on malaria in Myanmar. While the model uses data from the 3MDG reports, the analysis does not reflect any specific organisation or an average of the 3MDG programmes. Prices in local currency (Myanmar Kyat) were converted to US dollars using the mean interbank exchange rate for 2013 (1 US$ = 810 kyats) [12].

CHWs receive an initial training, which is typically supplemented by annual refresher trainings, the number of which depends on the duration of the project. CHWs are initially trained in malaria prevention and behavioural change communication, treatment of uncomplicated malaria according to national treatment guidelines, and patient registration. Some programmes include malaria as part of a wider package of services, this analysis relates to malaria-specific CHWs.

All ingredients identified in trainings were incorporated into the model including: travel costs for trainers, facilitators and trainees; food and accommodation, rental fees for training venue; expenses for stationary and learning aids; and fees for the trainers and facilitators. Total training costs were estimated depending on number of days and number of volunteers participating in the training.

Patient services include cost of commodities provided to CHWs to deliver malaria intervention and control to the community. The ingredients for patient services include: CHW kits, RDT and treatments for uncomplicated malaria (ACT plus single dose primaquine for *Plasmodium falciparum* malaria and chloroquine plus primaquine once a week over 8 weeks for *Plasmodium vivax* malaria). Anti-malarial medication and RDT are purchased separately by 3MDG and provided to implementing partners; the wholesale procurement price is used for these items. Data on the number of malaria tests performed in fever cases in the community and the number of positive cases were obtained from programme reports. The expected cost of patient services is calculated as a function of testing and positivity rates.

CHWs are usually monitored on a regular basis. In easy and medium accessibility areas a focal health centre model is used for CHW monitoring and support meetings. Once per month CHWs in the catchment area of a health centre meet to receive supplies, feed back data and receive general supervision. The proportion of CHWs attending monthly meetings is assumed to be 80 %. Supervisors also visit the villages quarterly to evaluate the CHWs’ work and to solve any problems the CHWs encounter. The ingredients for this activity include: travel cost for volunteers and supervisors; food and accommodation allowance; rental fee for meeting venues; and per diems for both volunteers and supervisors. In difficult and very difficult to access areas, field supervision trips to CHWs are performed by a mobile health team every 6 weeks as it is too burdensome for CHWs to travel to the nearest health facility. The teams provide on-site training and home visits of patients to evaluate the quality of the services performed and monitoring of correct use of resources. There are three members in a mobile supervision team and at least three villages are supervised in one trip, requiring a total of 4 days per supervision trip. In addition the daily cost of transportation is raised incrementally with remoteness category. The ingredients for this field supervision include travel cost for round trips and food, accommodation and salaries for malaria field supervisors.

Annual management cost for each CHW was calculated based on a hypothetical mid-sized organisation supporting 55 CHWs in five townships. For this model, all programme management is assumed to be undertaken by Myanmar nationals (rather than more expensive international staff). A 10 % overhead cost was applied to all cost centres except incentives to reflect office and utility costs. Incentives were included either as monthly fixed costs or according to performance of CHWs as detailed in the scenario analysis.

Opportunity costs of CHW time were estimated to explore full economic cost of CHW programme. The time contributed by CHW for malaria program were estimated and quantified the monetary value by multiplying average monthly salary in Myanmar US$ 180 per month [13]. These cost included time spent for training, patient service and time spent for being monitored by the supervisor. Time spent for each fever case by the CHW (24 min) was taken from a study in Ghana reporting the CHW working time in management of malaria in children [14].

### Sensitivity and scenario analysis

CHW programmes vary considerably between settings depending on a range of geographic, demographic, behavioural and programmatic factors. Scenario analyses were carried out to reflect the variation in field settings. Estimating annual cost of CHWs depends on the geographical location of the villages, epidemiology of malaria transmission, infrastructure such as accessibility of road condition, and transportation and other activities performed by the project.

Three dimensions of parameter variation are used in the scenario analysis (1) setting remoteness; (2) CHWs testing rate; and (3) financial incentives. These factors were identified as important to CHW programme costs during initial model development and through dialogue with programme managers and financial officers. The default number of RDT performed by each CHW is an average 135 per year, with an upper mean estimate of 250 tests and 20 tests as a lower estimate, reflecting a range of malaria testing in the CHWs reporting data (unpublished data, National Malaria Control Programme). The organizations implementing CHWs programmes in Myanmar use different incentive structures. The incentive schemes included here are (1) no incentive; (2) US$ 0.3 per RDT performed and US$ 5 per month; (3) US$ 0.5 per test and 0.5 per treated patient; (4) US$ 20 per month.

One-way sensitivity analysis was performed on all programme and economic parameters to assess their impact on the model. The upper and lower limits data are detailed in Table 1 and the results are presented in a tornado diagram.

The cost of financial remuneration (incentives) for CHW is a topic of interest to decision makers. The scenario analysis presents different incentive models and incentive amounts based on active CHW programmes in Myanmar. In addition univariate sensitivity analysis is presented on a range of values for monthly incentives and per test incentives. The changes in cost per CHW per year are explored as incentives varying from US$ 0 to 80 per month and US$ 0 to 4 per test. The incentive per test is more uncertain to the programme planner as it depends on the number of tests performed. This is included in the analysis, using testing rates sampled from a uniform distribution of the range in Table 1.

## Results

Rather than present a single general cost estimate this study presents a range of cost estimates in illustrative scenarios. The range of costs over the three dimensions of the scenario analysis (remoteness, testing rate and incentives) is US$ 966–2486 as detailed in Table 2. The range of annual costs across varying levels of remoteness, assuming no incentives and 135 tests per year is US$ 1061–2151. The range across varying levels of incentives, assuming medium accessibility and 135 tests per year is US$ 1266–1428. When varying the testing rate, assuming medium accessibility and no incentives the annual costs ranged from US$ 1103 to 1293. The value (opportunity cost) of CHW working time per year was US$ 130 assuming a subsistence wage rate. On this basis the annual economic cost is slightly higher than the financial cost.

**Table 2:** Annual cost of malaria CHW in Myanmar with variation in remoteness, incentives and testing rate

Location	Incentive	Annual CHW cost (US$)		
		Number of malaria RDT performed per CHW		
		Default (135)	Upper (250)	Lower (20)
Easily accessible area	No incentive	1061	1156	966
	US$ 0.3 per test and US$ 5 per month	1128	1281	976
	US$ 0.5 per test and US$ 0.5 per case	1161	1291	1032
	US$ 20 per month	1301	1396	1206
Medium accessible area	No incentive	1198	1293	1103
	US$ 0.3 per test and US$ 5 per month	1266	1418	1113
	US$ 0.5 per test and US$ 0.5 per case	1299	1428	1169
	US$ 20 per month	1428	1533	1343
Difficult to access area	No incentive	1986	2081	1891
	US$ 0.3 per test and US$ 5 per month	2954	2206	1901
	US$ 0.5 per test and US$ 0.5 per case	2087	2216	1957
	US$ 20 per month	2226	2321	2131
Very difficult to access area	No incentive	2151	2246	2056
	US$ 0.3 per test and US$ 5 per month	2219	2371	2066
	US$ 0.5 per test and US$ 0.5 per case	2252	2381	2122
	US$ 20 per month	2391	2486	2296

**Fig. 1:**
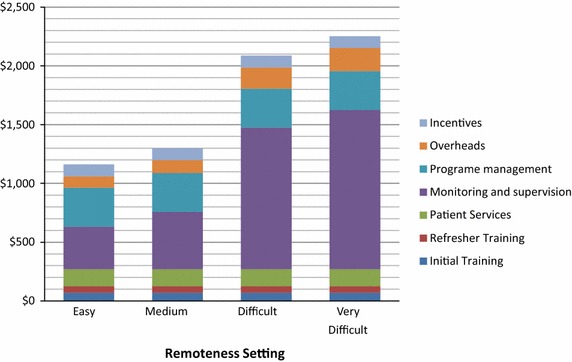
Breakdown of annual cost of malaria CHW in Myanmar by remoteness strata

The annual cost of CHWs varied most in different remoteness settings. Figure 1 presents the proportional contributions to cost from each cost centre within each remoteness scenario. The largest driver of CHW cost is monitoring and supervision (31–60 % of annual CHW cost). This is significantly larger in difficult and very difficult to access settings, where support is provided by mobile health teams. Other important determinants of cost include programme management (15–28 % of annual CHW cost) and patient services (6–12 % of annual CHW cost). Within patient services, malaria RDT are the major contributor to cost (64 % of patient service costs).

**Fig. 2:**
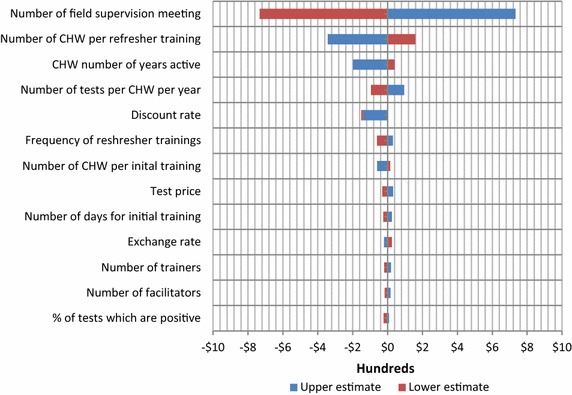
Variation in annual malaria CHW cost in Myanmar due to changes in a range of model parameters

Univariate sensitivity analysis of programme and economic variables (Fig. 2) finds that the number of field supervision meetings (assuming mobile field supervision) is the largest cost driver, followed by the average number of CHWs attending refresher trainings. Activities which are repeated over time and do not or might not benefit from economies of scale are expensive, such as mobile teams that regularly visits villages to provide support to one CHW at a time, or refresher trainings if only attended by a small number of CHWs.

**Fig. 3:**
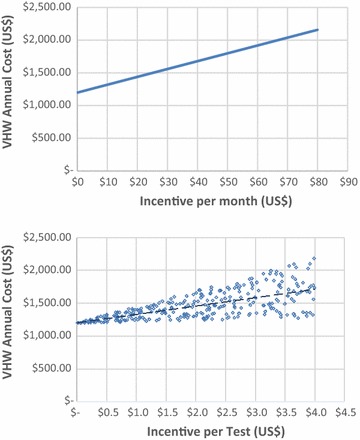
Variation in annual malaria CHW cost in Myanmar due to changes CHW financial incentives

The contribution of incentives to cost is, intuitively, highly sensitive to the size of the incentive. Figure 3 presents the rise from US$ 1253 to 2253 per CHW per year as incentives increase from US$ 0 to 80 per month and between US$ 1294 and 2207 per CHW per year as incentives increase from US$ 0 to 4 per test. Larger incentives might also increase testing rates implying higher expenditure for additional tests and treatments; this is not modelled here due to lack of relevant data.

## Discussion

This analysis provides guidance for comprehensive CHW programme budgeting, not only in the unit cost estimates, but through a better understanding of the key determinants of CHW programme costs.

This study estimates the annual cost of CHWs for malaria control and elimination in Myanmar to be between approximately US$ 1000 and 2500 per year depending on the remoteness, testing rate and financial incentives provided to the CHW. This is lower than the annual cost from a recent modelling study in an African context, with a mean estimated annual cost of US$ 3584 per CHW [15]. The main cost drivers are monitoring and supervision, programme management and patient services. Key programme variables are the number of field supervision visits per year; the average number of CHWs attending refresher trainings; the number of years a CHW is active for and the number of tests performed per year.

The cost of CHWs much depends on the geographical situation of the village. As CHWs aim to fill the gap of healthcare access inequity, they will be most needed in isolated villages in mountainous regions. The cost of maintaining CHWs in these areas will be high; field monitoring and supervision was identified as a key cost driver and in these peripheral areas is expensive, but perhaps also exactly where the service is most necessary. With minimal education and limited initial training, regular supervision is necessary to ensure the quality of CHWs services.

In this cost model, CHWs are volunteers and usually do not get a salary but depending on the specific programme and implementing organisation, they received incentives, either monetary or non-monetary such as, bags, umbrellas and torch lights printed with ministry of health logo. This paper outlines the contribution to programme cost of some of the incentive models currently being employed in Myanmar, but further work is needed to understand the impact of these incentives.

## Limitations

This cost analysis is based on financial reports of 3MDG and a review with programme managers, but not empirical measurement of CHWs cost. As such it is not an accurate reflection of the total economic costs of CHW activities. Only CHW activities relating to the management of febrile patients are included—diagnosing and treating malaria where appropriate and referring patients with danger signs. It did not include the cost of other malaria interventions that CHW might be involved with such as LLINs, IRS, IPT. In fact CHWs can be (and often are) active in care for other non-malarial causes of illness, implying higher costs on the one hand, but both the opportunity to share the costs with other programmes and with higher and more efficient returns on this investment.

## Conclusion

A better understanding of the costs of CHWs in different settings will improve policy makers’ and programme managers’ planning for malaria control and elimination in Myanmar. The costing model and its outputs will also be informative for economic evaluation of CHW activities that should account for factors such as the higher costs of maintaining CHWs in remote areas and the costs of different incentive schemes, as well as the varying returns on these investments.
